# IMPROVING COGNITIVE DECLINE FOR PERSONS LIVING WITH DEMENTIA: A RCT ART THERAPY BASED ON THE ETC FRAMEWORK

**DOI:** 10.1093/geroni/igad104.3743

**Published:** 2023-12-21

**Authors:** Heesu J Jeon

**Affiliations:** Adler University, Vancouver Campus, Vancouver, British Columbia, Canada

## Abstract

Abstract This randomized controlled trial explored the impact of Expressive Therapies Continuum (ETC)-based art therapy interventions for older adults with dementia living in a complex care home. The trial examined the efficacy of 10 art therapy intervention sessions over 4 months, comparing results with an active control group that participated in 10 art sessions. Pre- and post-assessments, including the Mini-Mental State Examination (MMSE), Clock Drawing Test (CDT), and the Older People’s Quality of Life (OPQOL-Brief) questionnaires, were conducted. Results demonstrated that participants displayed similar visual expression elements, shared preferences for specific media types, and equivalent ETC-based intervention entry levels. In addition, participants in the experimental group showed significant increases in OPQOL-Brief scores. Finally, MMSE-Language scores demonstrated statistically significant increases in the experimental group and a decrease in the control group. The current study aligns with previous research demonstrating that art therapy can improve cognitive functioning and enhance the quality of life in older adults living with dementia. It expands on previous research by providing new insights into expressive and stylistic elements, ETC entry-level, and media preferences for engaging in artmaking. Keywords: Art therapy, dementia, older adults, Expressive Therapies Continuum, cognitive functioning, quality of life

